# Relationship between lactate-to-albumin ratio and 28-day mortality in patients with exacerbation of chronic obstructive pulmonary disease admitted to the Intensive Care Unit

**DOI:** 10.1186/s40001-024-01867-8

**Published:** 2024-04-30

**Authors:** Jun Xie, Hui Liu, Qian He, Chong Li

**Affiliations:** Department of Respiration, First People’s Hospital of Changzhou, Third Affiliated Hospital of Soochow University, Juqian Road No.185, Changzhou, 213003 China

**Keywords:** AECOPD, Lactate to albumin ratio, 28-day mortality, Intensive Care Unit

## Abstract

**Aim:**

To explore the predictive value of lactate-to-albumin ratio (LAR) on 28-day mortality in patients with exacerbation of chronic obstructive pulmonary disease (AECOPD) admitted to the Intensive Care Unit (ICU).

**Methods:**

According to ICD-9 and ICD-10 diagnosis codes, patients diagnosed with AECOPD in the Medical Information Mart for Intensive Care IV (v.2.2) database were selected. The primary endpoint was 28-day mortality after ICU admission. We used receiver operating characteristic (ROC) curve, Kaplan–Meier (K–M) survival curve, logistic regression analyses and subgroup analysis to assess predictive power of LAR.

**Results:**

606 patients were included in this study. The 28-day mortality was 29.7%. The area under the ROC curves (AUC) for LAR were 0.641 [95% confidence interval (CI) 0.592–0.689], which was comparable with OASIS (AUC: 0.662; 95% CI 0.616–0.709; *p* = 0.471) and SOFA (AUC: 0.660; 95% CI 0.612–0.708; *p* = 0.500). The cutoff value of LAR was 0.645 by ROC curve. The high-LAR group showed a bad prognosis in K–M analysis (*p* < 0.001). Multivariate logistic regression shown that LAR was significantly associated with a poor outcome (odds ratio: 1.77; 95% CI 1.16–2.71; *p* = 0.008). Subgroup analysis showed no significant interaction of LAR with each subgroup (*p* for interaction: 0.175–0.775).

**Conclusion:**

LAR is a rational and easily accessible marker, which is remarkably associated with 28-day mortality in ICU patients with AECOPD.

## Introduction

It is well recognized that chronic obstructive pulmonary disease (COPD) is becoming a major global health concern. In 2019, the global prevalence of COPD among people aged 30–79 years was 10.3% [1]. COPD is the third leading cause of mortality globally and the total number of deaths from COPD reached 3.23 million in 2019 [2, 3]. Due to its high prevalence and chronicity, patients suffer from frequent hospitalizations due to acute exacerbations [4]. Acute exacerbation in COPD (AECOPD) is a significant risk factor contributing to hospitalization and mortality in affected COPD patients [5]. Thus, meaningful markers associated with mortality in patients with AECOPD admitted to the ICU are needed.

Lactate is an important metabolite product of the glycolytic process. Previous studies have uncovered lactate has the strongest relation with mortality in critically ill patients [6, 7]. Elevation of lactate is associated with hypoxia and hypoperfusion and with the use of some medications [8]. Chronic hypoxia is one of the most important features of COPD. During hypoxia conditions, increased glycolysis causes a surge in lactate production [9]. In addition, as a consequence of chronicity, malnutrition is also a common feature among patients with COPD [10]. The diagnosis of malnutrition was based on albumin level of less than 3.5 g/dL. In patients admitted to ICU, low serum albumin levels (< 3 g/dL) increased in-hospital mortality than those with high serum albumin levels (< 3 g/dL) with odds ratio of 1.51 [11]. Therefore, lactate and albumin might be associated closely with outcomes for patients with COPD. In recent years, lactate to albumin ratio (LAR) have been proposed to be related with clinical outcome in critically ill patients [12], spontaneous subarachnoid hemorrhage [13], and community-acquired pneumonia [14]. However, the prognostic value of LAR in patients with AECOPD admitted to the ICU remains unclear.

In the present study, we explored the potential relation between LAR and 28-day mortality in patients with AECOPD admitted to the ICU, and compared its predictive power with other commonly used clinical risk scores.

## Materials and methods

### Patients selection

**Fig. 1:**
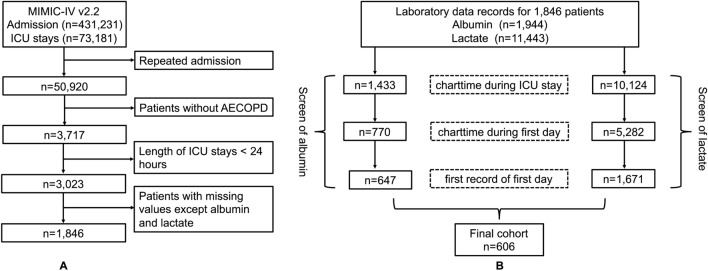
The flowchart of the screening process. **A** Initial screening. **B** Final screening

It is a retrospective cohort study. Medical Information Mart for Intensive Care IV (MIMIC-IV) database is a single center data from Beth Israel Deaconess Medical Center which contains information relating to patients admitted to critical care units between 2008 and 2019. The latest version is released at 2023–01–06, with version number v2.2. We used ICD-9 codes (491, 492 or 496), and ICD-10 codes (J41–J44) to defined COPD. Moreover, any of the three COPD codes listed in the primary diagnosis field as AECOPD according to published study [15]. The exclusion criteria were as follows: repeated admission, ICU stay time less than 24 h and patients with missing data. One of the authors (QH) had been given permission to extract data from this database after completing the database usage examination (ID: 49872601). Due to the relatively large number of missing data for lactate and albumin, we divided screening process into two portions for more clearly as shown in Fig. 1. Data from MIMIC-IV are publicly available, thus this study was exempted by the ethics committee of the First People’s Hospital of Changzhou.

### Variable extraction

Information, including demographic (age, gender), first-day severity of illness [sequential organ failure assessment (SOFA), Oxford Acute Severity of Illness (OASIS), Glasgow coma scale (GCS)], comorbidities (myocardial infarct, congestive heart failure, diabetes, renal disease, malignant cancer, delirium, sepsis, tobacco status), first-day laboratory tests [arterial blood gases (pH, arterial carbon dioxide tension, arterial oxygen tension, base excess), white blood cell, platelet, red blood cell distribution width, hematocrit, hemoglobin, creatinine, glucose, potassium, sodium, lactate, and albumin], first-day vital signs (hear rate, respiratory rate, mean arterial blood pressure, SpO_2_), status of mechanical ventilation, survival time were obtained. If patients had multiple measurements for some variables at first day on ICU admission, only the first value was used. Distribution normality was evaluated using Shapiro–Wilk normality test. Continuous variables were described as means and standard or median (25th–75th percentile), and categorical variables as proportions. The main outcome was defined as 28-day mortality after ICU admission.

### Statistical analysis

Differences between different groups in baseline data analysis were compared using *t*-test or Wilcoxon rank-sum test for continuous variables, and the chi-square test for categorical variables. The receiver operating characteristic (ROC) curves were used to predictive analysis and identify cut-off values, and the area under the ROC curve (AUC) was calculated. According to cutoff value of LAR, patients were divided into high LAR and low LAR. Significance of survival analysis was performed by K–M curve with log-rank test. Univariate and multivariate logistic regression models were performed to explore the relationship between LAR and 28-day mortality in AECOPD patients. Factors with a *p*-value of less than 0.05 in the univariate analysis were entered in the multivariate analysis. Stratified and interaction analyses were applied to investigate whether LAR had any effect in different comorbidities.

All data were extracted by Navicat Premium 16.1.3 software. Statistical analysis was performed using R language. *P*-value < 0.05 was considered as statistically significant.

## Results

### Patients characteristics

A total of 606 patients were included in the study. Figure 1 shows the screening process in a flow diagram. The median age of the study population was 72 years old. In the total study population, male subjects accounted for 56.1%. Compared with the survivors, the death group were older and had more severe SOFA and OASIS scores, and a higher proportion of mechanical ventilation. In addition, higher levels of white blood cell, red blood cell distribution width, hemoglobin, creatinine, glucose, and LAR were observed in the death group. More details are available in Table 1.

**Table 1:** Baseline characteristics between survivors and non-survivors

Variable	Total (*N* = 606)	Survivors (*N* = 426)	Death (*N* = 180)	*p*-value
Demographics				
Gender				
Male	340 (56.1)	239 (56.1)	101 (56.1)	1
Female	266 (43.9)	187 (43.9)	79 (43.9)	
Age (years)	72.00 [63.00, 79.00]	70.00 [61.00, 78.00]	74.50 [66.00, 82.25]	< 0.001
Severity of illness				
SOFA	7.00 [4.00, 9.00]	6.00 [4.00, 9.00]	8.00 [6.00, 11.00]	< 0.001
OASIS	37.00 [31.00, 43.00]	35.50 [30.00, 42.00]	41.00 [35.00, 48.00]	< 0.001
GCS	15.00 [13.00, 15.00]	15.00 [13.25, 15.00]	15.00 [10.75, 15.00]	0.393
Comorbidities				
Myocardial infarct	135 (22.3)	87 (20.4)	48 (26.7)	0.114
Congestive heart failure	237 (39.1)	165 (38.7)	72 (40.0)	0.841
Diabetes	180 (29.7)	124 (29.1)	56 (31.1)	0.692
Renal disease	138 (22.8)	95 (22.3)	43 (23.9)	0.749
Malignant cancer	95 (15.7)	55 (12.9)	40 (22.2)	0.006
Delirium	61 (10.1)	45 (10.6)	16 (8.9)	0.632
Tobacco status	155 (25.6)	107 (25.1)	48 (26.7)	0.766
Sepsis	500 (82.5)	345 (81.0)	155 (86.1)	0.161
Laboratory results				
WBC (10^9^/L)	12.10 [8.30, 17.10]	11.45 [8.20, 16.12]	13.80 [9.28, 19.92]	0.001
Platelet (10^9^/L)	216.00 [151.50, 293.50]	215.00 [155.25, 294.00]	217.00 [148.50, 283.25]	0.63
RDW (%)	15.10 [14.00, 16.40]	14.90 [14.00, 16.08]	15.60 [14.20, 17.10]	0.001
Hematocrit (%)	34.60 [30.02, 39.88]	35.05 [30.50, 40.40]	34.05 [29.30, 38.82]	0.111
Hemoglobin (g/dL)	11.20 [9.80, 13.00]	11.30 [9.90, 13.20]	10.95 [9.60, 12.43]	0.044
Creatinine (mg/dL)	1.20 [0.80, 1.80]	1.10 [0.80, 1.78]	1.30 [0.90, 1.90]	0.044
Glucose (mg/d)	135.00 [106.00, 178.00]	131.50 [105.00, 172.00]	153.00 [116.75, 198.25]	0.001
Potassium (mmol/L)	4.40 [3.90, 4.90]	4.30 [3.90, 4.90]	4.50 [3.90, 5.00]	0.05
Sodium (mmol/L)	138.00 [135.00, 141.00]	138.00 [135.00, 141.00]	139.00 [135.00, 142.00]	0.372
PaO_2_	100.00 [76.00, 168.00]	101.50 [77.00, 176.75]	97.00 [75.00, 163.25]	0.328
PaCO_2_	44.00 [38.00, 53.00]	43.00 [38.00, 52.75]	45.00 [37.00, 55.00]	0.35
pH	7.35 [7.27, 7.41]	7.36 [7.29, 7.41]	7.33 [7.23, 7.39]	< 0.001
Base excess	0.00 [− 5.00, 2.00]	0.00 [− 4.00, 2.00]	− 2.00 [− 7.00, 1.00]	< 0.001
LAR	0.58 [0.39, 0.91]	0.53 [0.36, 0.76]	0.72 [0.47, 1.25]	< 0.001
Vital signs				
HR (bpm)	91.00 [79.00, 107.00]	90.00 [79.00, 105.75]	95.00 [81.75, 109.25]	0.041
RR (bpm)	20.00 [16.00, 24.00]	20.00 [16.00, 24.00]	20.00 [17.00, 25.00]	0.084
MBP (mmHg)	80.00 [69.00, 94.00]	81.00 [70.00, 95.00]	77.00 [64.00, 91.00]	0.005
SpO_2_ (%)	97.00 [94.00, 100.00]	98.00 [95.00, 100.00]	97.00 [93.00, 100.00]	0.102
Mechanical ventilation	423 (69.8)	282 (66.2)	141 (78.3)	0.004

*SOFA* sequential organ failure assessment, *OASIS* oxford acute severity of illness score, *GCS* Glasgow coma scale, *WBC* white blood cell, *RDW* red blood cell distribution width, *LAR* lactate albumin ratio, *HR* heart rate, *RR* respiratory rate, *MBP* mean arterial blood pressure

### ROC curve analysis and Kaplan–Meier curve

**Fig. 2:**
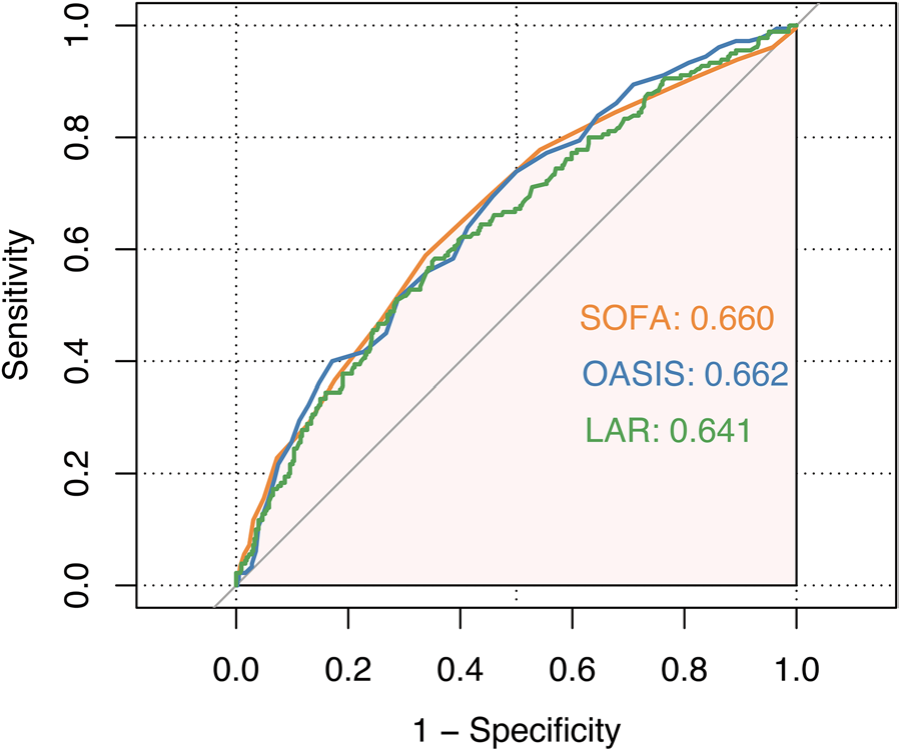
ROC comparison for predicting 28-day mortality among LAR, SOFA and OASIS

We plotted ROC curves for the three indicators of LAR, SOFA and OASIS for predicting 28-day mortality in ICU patients with COPD. The detailed information in Fig. 2 is listed in Table 2. The AUC of LAR (0.641; 95% CI 0.592–0.689) was comparable with OASIS (0.662; 95% CI 0.616–0.709; *p* = 0.471) and SOFA (0.660; 95% CI 0.612–0.708; *p* = 0.500).

**Table 2:** Information of receiver operating characteristic curves in Fig. 2

Variables	AUC (crude)	95% CI	Cut-off	Sensitivity	Specificity	*p*-value
LAR	0.641	0.592–0.689	0.645	0.583	0.646	Ref
SOFA	0.660	0.612–0.708	7.500	0.589	0.662	0.500
OASIS	0.662	0.616–0.709	35.500	0.739	0.500	0.471

**Fig. 3:**
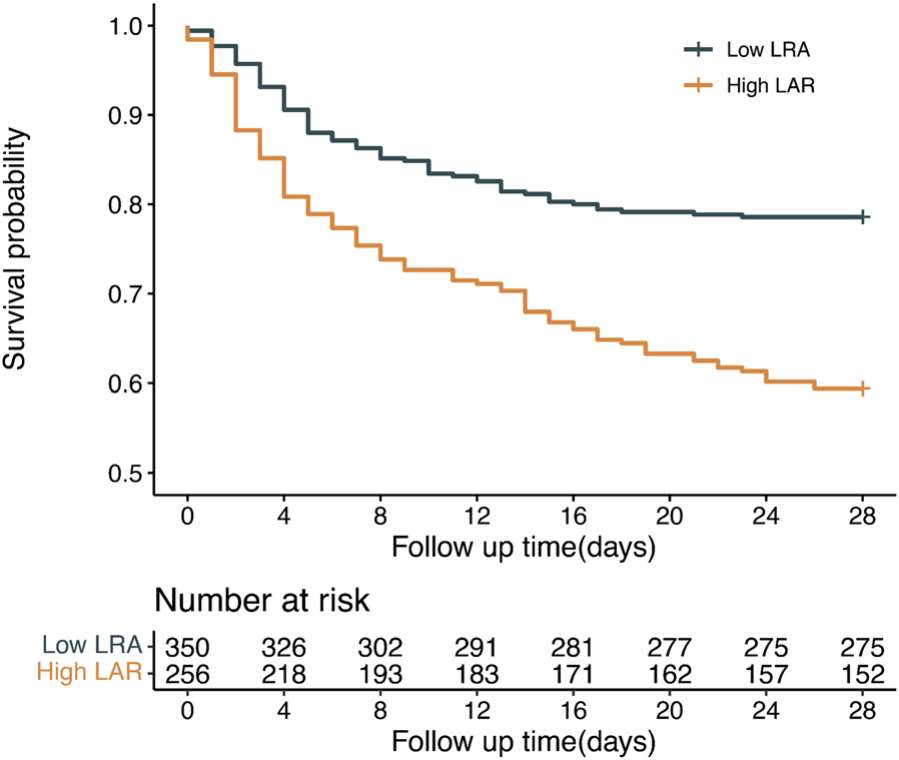
Kaplan–Meier survival curves for 28-day of patients with AECOPD admission to ICU

LAR cutoff value was < 0.645 by ROC analysis. According to the optimal cut-off value, patients were divided into low LAR group (LAR < 0.645, *n* = 350) and high LAR group (LAR ≥ 0.645, *n* = 256). As shown in Fig. 3, survival curve indicated that the prognosis in the high LAR group was significantly poorer than that in the low LAR group (*p* < 0.001).

### Univariate and multivariate logistic regression analyses of LAR and 28-day mortality

Table 3 shows the results of univariate and multivariate logistic regression analyses. A total of fifteen variables (age, SOFA, OASIS, GCS, white blood cell, red blood cell distribution width, hemoglobin, glucose, pH, base excess, LAR, mean arterial blood pressure, SpO_2_, malignant cancer, and status of mechanical ventilation) were statistically significant (*p* < 0.05) in the univariate logistic regression analysis. These fifteen variables were further included in the multivariate logistic regression analysis. Multivariate analyses show that compared with low group, high group had higher risk of 28-day mortality (OR: 1.77; 95% CI 1.16–2.71; *p* = 0.008).

**Table 3:** Univariate and multivariate logistic regression analysis of factors associated with 28-day mortality in patients with AECOPD

Variable	OR (univariable)	OR (multivariable)
Gender		
Male	Ref	
Female	1.00 (0.70–1.42, *p* = 0.999)	
Age (years)	1.04 (1.02–1.05, *p* < 0.001)	1.04 (1.02–1.06, *p* < 0.001)
Severity of illness, points		
SOFA	1.17 (1.11–1.23, *p* < 0.001)	1.08 (1.01–1.16, *p* = 0.028)
OASIS	1.07 (1.05–1.09, *p* < 0.001)	1.03 (1.00–1.06, *p* = 0.096)
GCS	0.94 (0.89–0.98, *p* = 0.010)	1.00 (0.94–1.07, *p* = 0.916)
Comorbidities		
Myocardial infarct	1.42 (0.94–2.13, *p* = 0.092)	
Congestive heart failure	1.05 (0.74–1.51, *p* = 0.770)	
Diabetes	1.10 (0.75–1.61, *p* = 0.622)	
Renal disease	1.09 (0.72–1.65, *p* = 0.670)	
Malignant cancer	1.93 (1.23–3.03, *p* = 0.004)	2.49 (1.49–4.16, *p* < 0.001)
Delirium	0.83 (0.45–1.50, *p* = 0.532)	
Tobacco status	1.08 (0.73–1.61, *p* = 0.690)	
Sepsis	1.46 (0.89–2.37, *p* = 0.131)	
Laboratory results		
WBC (10^9^/L)	1.04 (1.02–1.06, *p* = 0.001)	1.04 (1.01–1.06, *p* = 0.011)
Platelet (10^9^/L)	1.00 (1.00–1.00, *p* = 0.668)	
RDW (%)	1.16 (1.06–1.26, *p* = 0.001)	1.18 (1.06–1.31, *p* = 0.003)
Hematocrit (%)	0.98 (0.96–1.01, *p* = 0.123)	
Hemoglobin ((g/dL)	0.93 (0.86–1.00, *p* = 0.040)	0.98 (0.90–1.07, *p* = 0.685)
Creatinine (mg/dL)	1.02 (0.92–1.14, *p* = 0.678)	
Glucose (mg/d)	1.00 (1.00–1.00, *p* = 0.044)	1.00 (1.00–1.00, *p* = 0.260)
Potassium (mmol/L)	1.14 (0.95–1.36, *p* = 0.155)	
Sodium (mmol/L)	1.02 (0.99–1.05, *p* = 0.131)	
pH	0.04 (0.01–0.21, *p* < 0.001)	0.09 (0.01–1.01, *p* = 0.051)
PaO_2_	1.00 (1.00–1.00, *p* = 0.195)	
PaCo_2_	1.01 (1.00–1.02, *p* = 0.212)	
Base excess	0.95 (0.92–0.98, *p* < 0.001)	1.03 (0.98–1.08, *p* = 0.204)
LAR		
< 0.645	Ref	
≥ 0.645	2.55 (1.78–3.64, *p* < 0.001)	1.77 (1.16–2.71, *p* = 0.008)
Vital signs		
HR (bpm)	1.01 (1.00–1.02, *p* = 0.086)	
RR (bpm)	1.02 (0.99–1.05, *p* = 0.213)	
MBP (mmHg)	0.99 (0.98–1.00, *p* = 0.003)	0.99 (0.98–1.00, *p* = 0.040)
SpO_2_ (%)	0.96 (0.92–0.99, *p* = 0.013)	0.96 (0.92–1.00, *p* = 0.031)
Mechanical ventilation	1.85 (1.23–2.77, *p* = 0.003)	1.42 (0.82–2.49, *p* = 0.214)

*OR* odds ratio, *SOFA* sequential organ failure assessment, *OASIS* oxford acute severity of illness score, *GCS* Glasgow coma scale, *WBC* white blood cell, *RDW* red blood cell distribution width, *LAR* lactate albumin ratio, *HR* heart rate, *RR* respiratory rate, *MBP* mean arterial blood pressure, *MV* mechanical ventilation

### Subgroup analysis

**Fig. 4:**
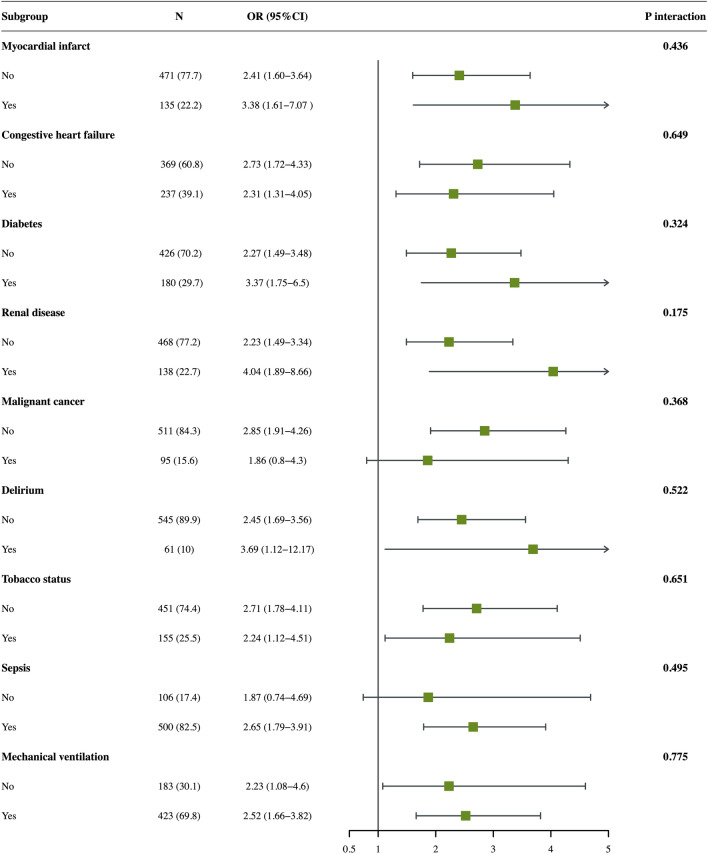
Subgroup analysis for the relationship between LAR and risk of 28-day mortality

Figure 4 provided the results of stratified analysis in different subgroups, including myocardial infarct, congestive heart failure, diabetes, renal disease, malignant cancer, delirium, sepsis, tobacco status, and mechanical ventilation. In all subgroup excluding patients with malignant cancer or sepsis, high LAR level was related to significantly elevated risk of 28-day mortality (*p*-value < 0.05). There were no interactions in all subgroups (*p*-value > 0.05).

## Discussion

Given that populations are rapidly aging worldwide, age-related diseases like COPD are increasing in frequency [16]. It imposes a large burden on the affected individual and society, even in the developed countries [17]. Therefore, it is particularly important to find new prognostic indicators to improve the current situation. In this study, we found that the predictive value for 28-day mortality of the LAR was revealed to be the same as that of OASIS and SOFA in patients with AECOPD admitted to the ICU through ROC curves and K–M curves. Then, univariate and multivariate logistic regression analyses suggested that LAR was an independent risk factor affecting prognosis. Furthermore, subgroup analyses suggested that the relationships between LAR and 28-day mortality in different subgroups were stable in all groups excepted malignant cancer.

It is well known that the risk of short-term death is increased with higher lactate concentrations in critically ill patients [18]. However, the level of lactate is susceptible to many factors, such as drug, liver dysfunction and regional ischemia [12]. In a systematic review, Zachary et al. reported that salbutamol was one of two most commonly identified agents which cause medication-induced lactate level elevations [19]. Patients with normal level of lactate remain predisposed to potential high mortality risks [20]. Thus, lactate alone could result in the bias of prediction accuracy, especially in patients with AECOPD.

To our knowledge, this is the first time that the role of LAR in AECOPD has been reported. For each unit increase in LAR, risk of 28-day mortality increased 1.38-fold. This relation can tentatively be explained as follows. Due to progressive airflow limitation and emphysematous destruction of the pulmonary capillary bed, ventilation/perfusion mismatch contributors to hypoxemia [21]. Thus, hypoxemia is an elementary pathophysiological disorder associated with AECOPD. More than 80% of patients with advanced disease participating in the National Emphysema Treatment Trial were using some form of oxygen therapy [22]. Low oxygen delivery states lead to hyperlactatemia [23]. Moreover, increased respiratory rate leads to respiratory muscle fatigue and thus exacerbate increased lactate levels. Malnutrition in patients with COPD is associated with cachexia, sarcopenia, and weight loss [24]. Previous studies have estimated that 25–40% of COPD patients are underweight while 35% of patients have severely low fat-free mass index (FFMI) [25]. Low FFMI increased the risk of mortality 17 times. Combined with the above reasons, LAR could reflect the physiology changes caused by different mechanisms in patients with AECOPD.

There are strengths and limitations to our study. On one hand, LAR is a simple blood test. It is easy to promote in primary hospitals. Our study demonstrated that LAR was a moderate prognostic factor to predict 28-day mortality in patients with AECOPD. On the other hand, cutoff values may be helpful for risk stratification and early interventions. Apart from these advantages, our study was challenged by a rather large number of missing data for lactate and albumin at first day on ICU admission. As shown in Fig. 1B, missing data may be resolved effectively if LAR is studied as recorded during hospitalization, but early prediction power will be lost. Second, LAR is a time varying variable that can change over the course [26]. The study results apply only to patients had lactate and albumin records at first day on ICU admission. Longitudinal data would be needed. Last, other factors which could not be collected, including pulmonary function and drug use before hospital admission (like metformin), and severity of AECOPD, may have impacted on results.

## Conclusion

According to the current research, LAR is remarkably associated with 28-day mortality in ICU patients with AECOPD. The mechanisms under this relation are in line with physiopathological processes of COPD. LAR is an important predictor of 28-day mortality, which is simple to acquire and to monitor.

## Data Availability

Data of this study were publicly available, which could be download from https://mimic.mit.edu. One of the authors (QH) had been given permission to extract data from this database after completing the database usage examination (ID: 49872601).
